# Supplementary material for: Actionable Predictive Factors of Homelessness in a Psychiatric Population: Results from the REHABase Cohort Using a Machine Learning Approach
Source: Int J Environ Res Public Health. 2022 Sep 27;19(19):12268. doi: 10.3390/ijerph191912268 (PMC9565981; doi:10.3390/ijerph191912268)
Supplement: Supplementary file 1 [file ijerph-19-12268-s001.zip › ijerph-1892287-supplementary.pdf]

Actionable predictive factors of homelessness in a psychiatric population: results from the REHABase cohort using a machine learning approach

Supplementary files

Supplementary Table S1. *Selected potential predictor of homelessness.*

Name	*Type*	*Number of unique values*	*Size of the population*	*Missing values* *(Total population size : 3416)*
**Gender / Sex**	Categorical	2	3415	0.03%
**Diagnosis (DSM-5)**	Categorical	20	3404	0.35%
**Psychotropic medication (INI/DCI)**	Categorical	80	3403	0.38%
**Age of 1st symptoms**	Numerical	783	2913	14.7%
**GAF**	Numerical (1-100 scale)	68	2769	18.9%
**CGI**	Numerical (1-7 scale)	7	2761	19.17%
**MARS**	Numerical (0-10 scale)	11	1645	51.8%
**Insight (BIS)**	Numerical (0-12 scale)	25	1691	50.5%

Supplementary analysis 1 – *Analysis of the robustness of estimates of homelessness predictors' importance.*

Homelessness predictors importance was estimated using decision classification and regression trees (Classification and Regression Trees : CART [20,21]) with the fitctree function of the Matlab R2018a statistics and machine learning toolbox using the 'interaction-curvature' option for predictors selection and surrogate splits to handle missing data (option 'surrogate', 'all'). Then unbiased estimator of predictors' importance can be calculated by summing changes in the risk due to splits the tree on every predictor and dividing the sum by the number of branch nodes [23]. In this study we are confronted with a substantial but largely unbalanced data set. This is why we chose to estimate the importance of the predictors on balanced groups resampling the data set many times.

Each choice of parameter is made with the aim of optimizing the benefit/risk ratio. The risk being an overestimation of the importance of a variable, the benefit being to be sensitive enough to identify all the variables of interest. As these choices can be discussed, we analyze here the robustness of our results according to modifications of our analysis parameters.

a) Classification performance estimates.

Performance optimization is not the objective of this analysis which aims at estimating in an unbiased way the relative importance of the different potential predictors. Performance optimization can be done in a second step using hyperparameters optimization and detailed analysis of the most relevant variables. Nevertheless, for the sake of completeness, we estimate and report the classification performances obtained from this architecture and this dataset. 500 estimates were made by resampling the data. Each training is performed on a balanced set of size N=640, the validation scores being estimated for each resampling on 2771 individuals for the specificity estimation, 5 individuals being excluded from the training set for the sensitivity estimations.

The classification performances are shown in fig. S1-A. The p-values are obtained by analyzing each estimate with a sign test under the null hypothesis that the median performance is not different from 50%. The importance estimates of the different predictors, reported in fig. S1-B, do not differ from the importance reported in the paper despite the exclusion of 5 homeless individuals at each resampling for sensitivity estimates. These results can be improved by optimizing hyperparameters and especially by limiting the complexity of the classification trees. But this impacts the quality of the estimate of the predictors importance which is our measure of interest.

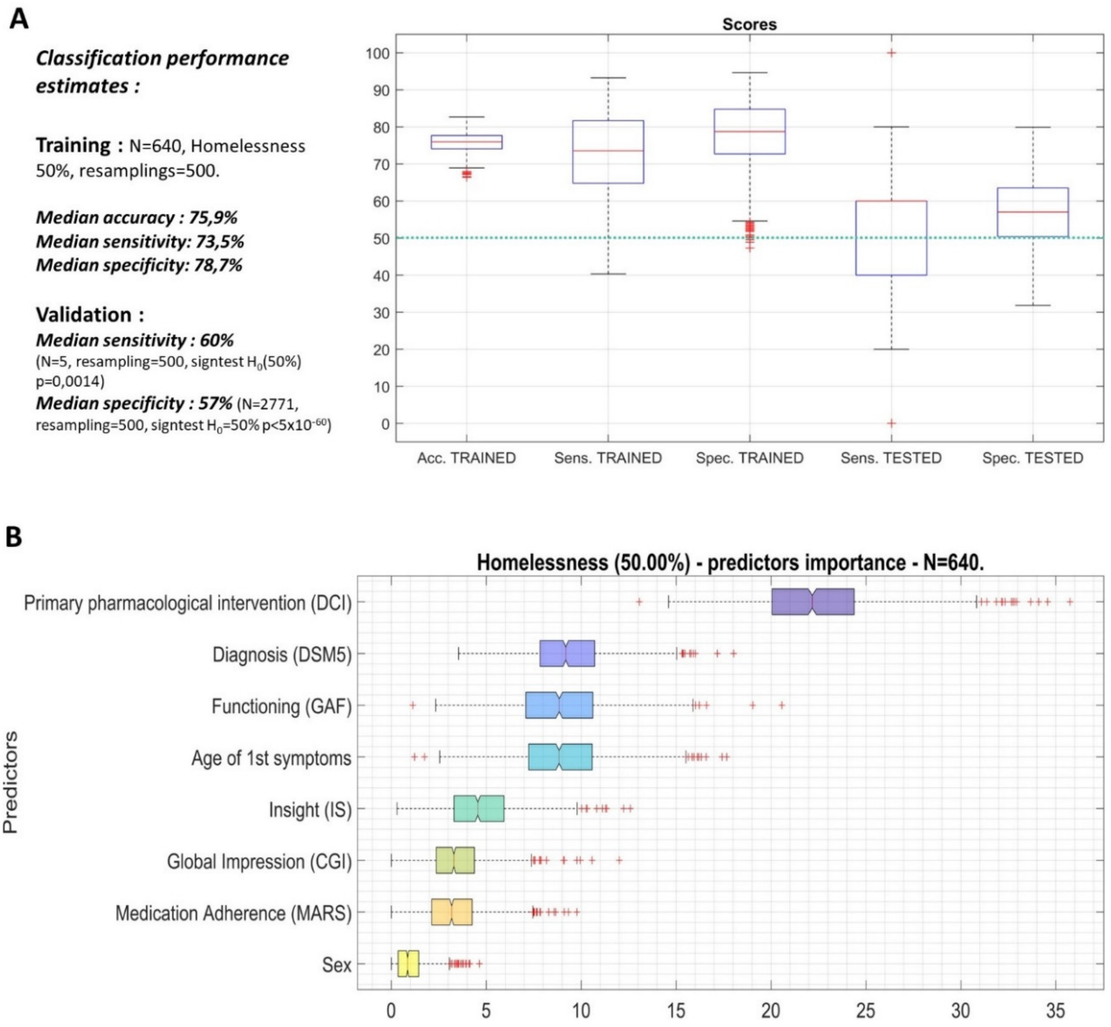
A
Classification performance estimates :
Training : N=640, Homelessness 50%, resamplings=500.
Median accuracy : 75,9%
Median sensitivity: 73,5%
Median specificity: 78,7%
Validation :
Median sensitivity : 60%
(N=5, resampling=500, signtest H_0(50%) p=0,0014)
Median specificity : 57% (N=2771, resampling=500, signtest H_0=50% p<5x10^{-60})
Scores
100
90
80
70
60
50
40
30
20
10
0
Acc. TRAINED
Sens. TRAINED
Spec. TRAINED
Sens. TESTED
Spec. TESTED
B
Homelessness (50.00%) - predictors importance - N=640.
Predictors
Primary pharmacological intervention (DCI)
Diagnosis (DSM5)
Functioning (GAF)
Age of 1st symptoms
Insight (IS)
Global Impression (CGI)
Medication Adherence (MARS)
Sex
0
5
10
15
20
25
30
35

Figure S1

b) **Unbalanced VS balanced data.**

In order to prevent the algorithms from converging to trivial solutions and to ensure that the importance of the predictors are estimated using the information of both populations in an equivalent way, the training was performed on balanced data sets. However, this has the disadvantage of greatly reducing the size of the training set, which may reduce the ability to identify relevant variables. Thus, an estimation of the importance of the predictors by maximizing the size of the training set (but with K-fold=200 cross-validation) was performed and is presented in Figure S2-A, compared to the original analysis presented in Figure S2-B.

The overall arrangement of predictors remains similar, except for the variable 'Age of 1st symptoms' which suggests a possible sampling bias. Nevertheless, the post-hoc analysis on this particular variable presented at the end of this document did not reveal a significant relationship with homelessness.

TEST UNBALANCED DATA

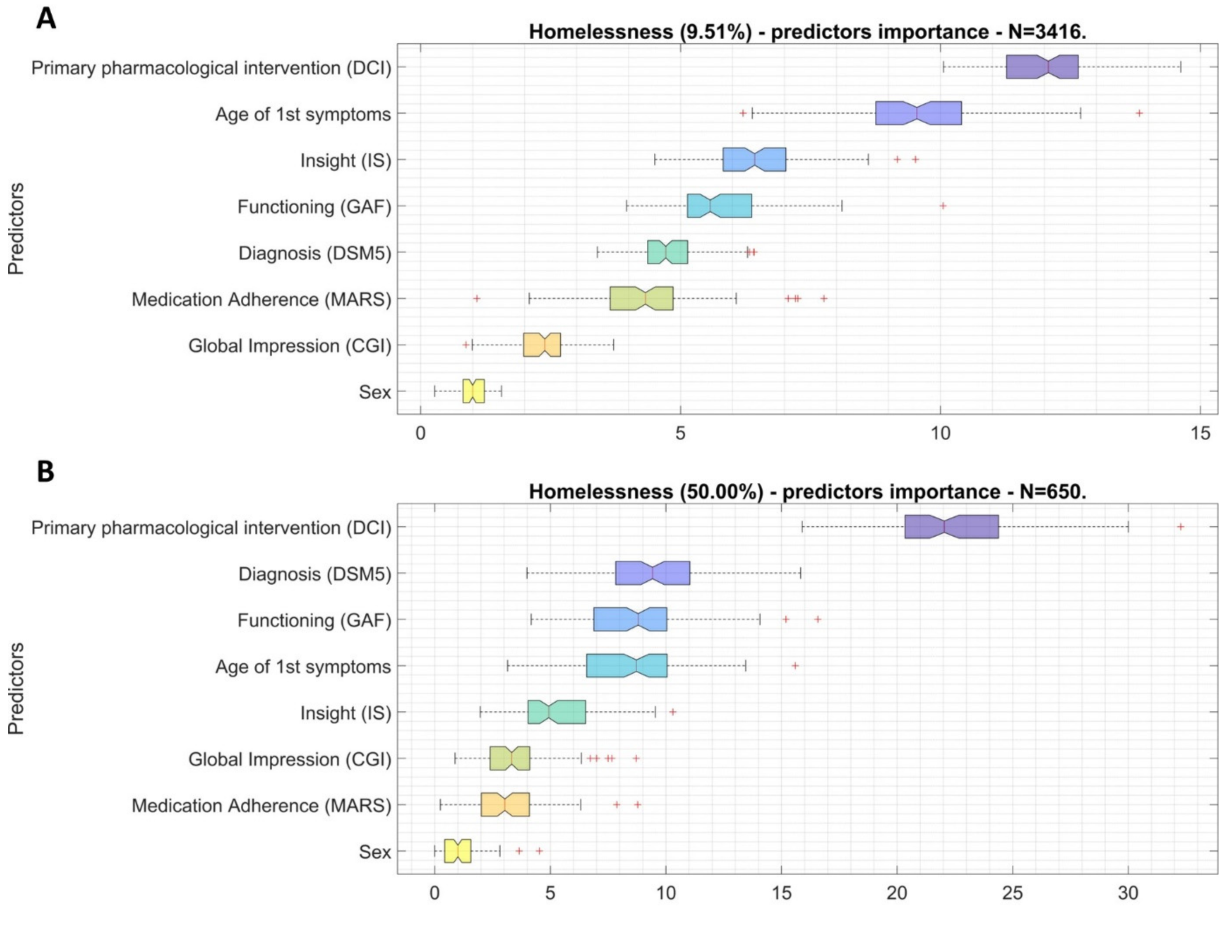

Figure S2

c) Robustness regarding missing data.

Specific procedures such as surrogate-splits were used in this study to overcome the problem of missing values. Nevertheless, as missing values can be numerous for some variables, alternative analyses with systematic exclusion of any subject with a missing value are presented here:

With balanced data in figure S3-B, compared to the original result in S3-A. With unbalanced data in S4-B compared to the analysis with full data in S4-A.

The overall arrangement of the data remains similar. The order of the variables discussed in the original analysis (gender and psychotropic medication) remaining the same.

TEST MISSING DATA ROBUSTNESS

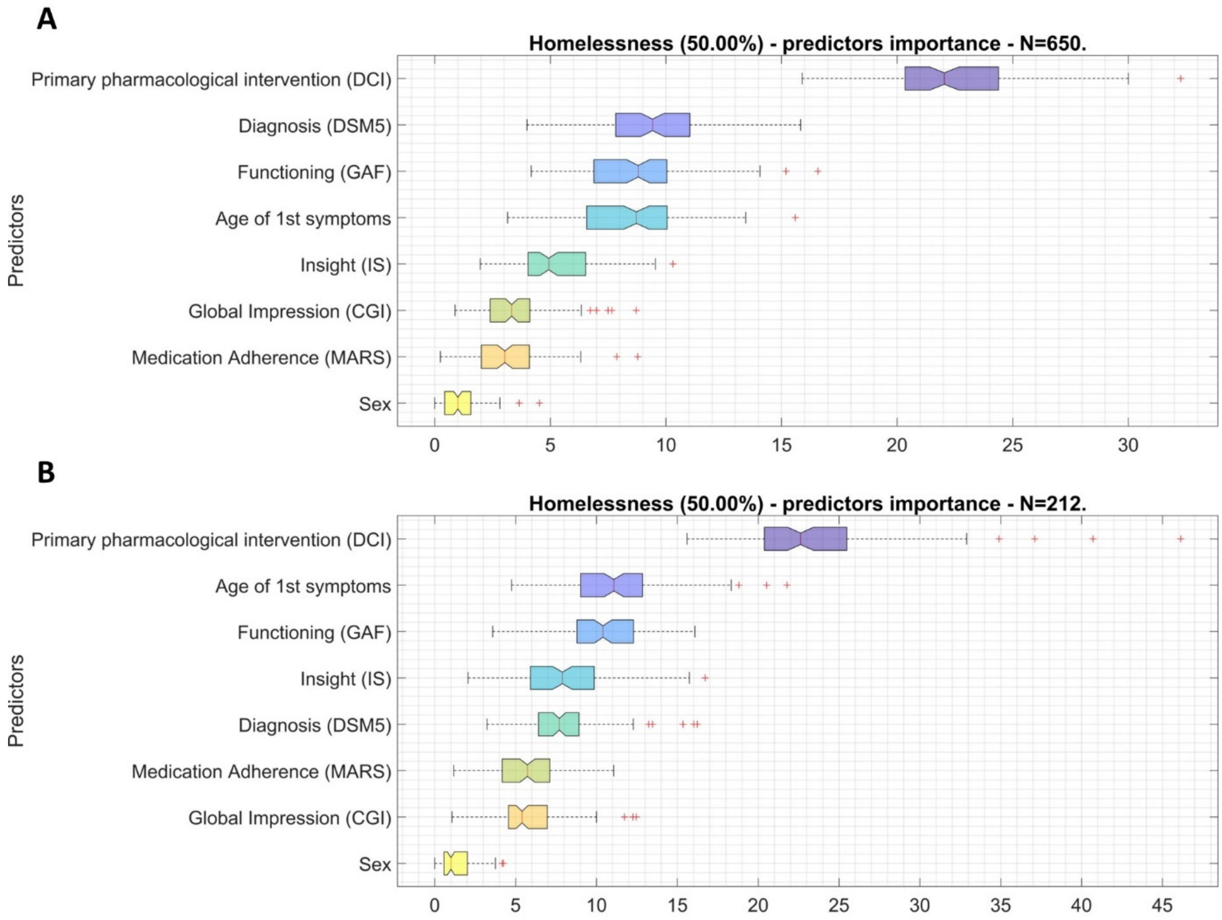

Figure S3

TEST MISSING DATA ROBUSTNESS
(unbalanced data)

A

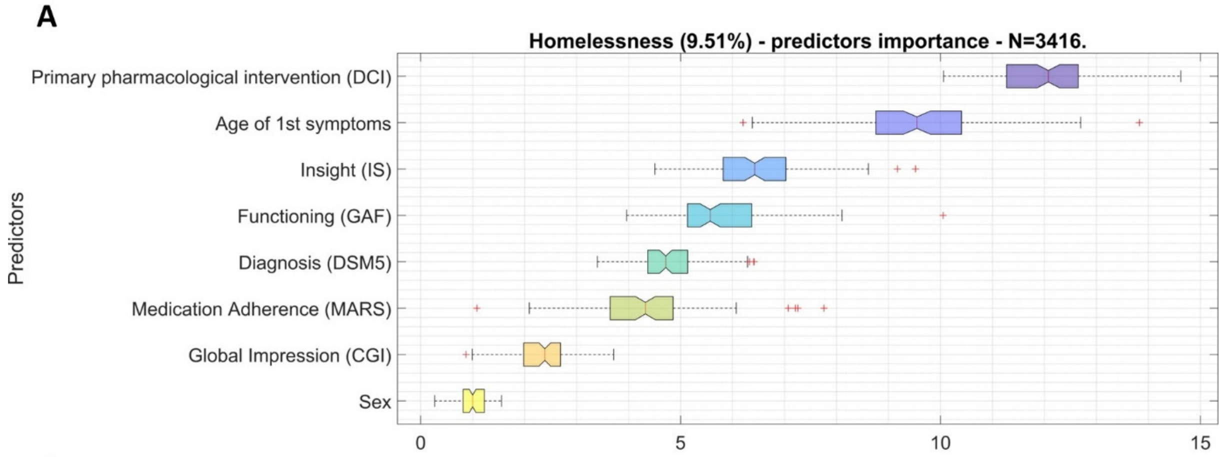

B

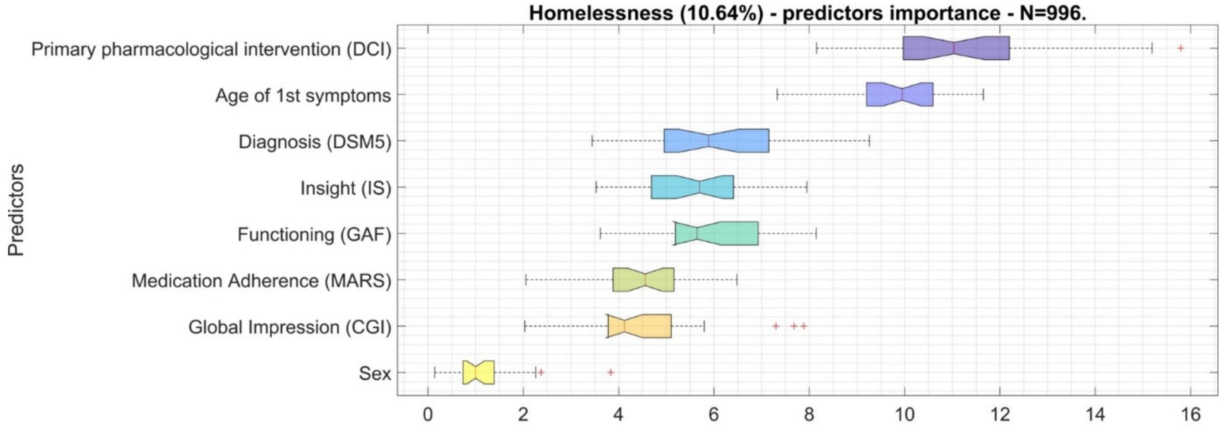

Figure S4

d) Robustness regarding the random predictor test.

An interesting analysis to challenge machine learning procedures is to add random variables of different natures to the dataset in order to verify the robustness of the method with non-informative data. Two new variables were added to the original set. A binary random variable (RandomBIN) and a random variable drawn on a uniform distribution (RandomUNIFORM) which will have a unique value for each subject in the database.

The binary random variable is expected to have the lowest predictive power and is useful for analyzing the predictive power of other binary variables such as sex. The uniform random variable is used to assess the risk of overlearning. Each value being unique, it is possible in overlearning to generate a classifier that will be able to classify all the individuals in a perfect way, but that will have null performances in the validation phase.

The estimates of predictor importance of the original procedure are presented in figure S5-A, and with the two added random variables in S5-B. As expected, the random variable RandomBIN had the lowest predictive power, but was not significantly lower than the gender variable. This highlights the questionable value of including gender information in prevention and psychosocial support policies. The RandomUNIFORM variable is well estimated with a high predictive power, but much lower than the psychotropic medication variable which remains the factor of primary importance.

TEST RANDOM VARIABLE

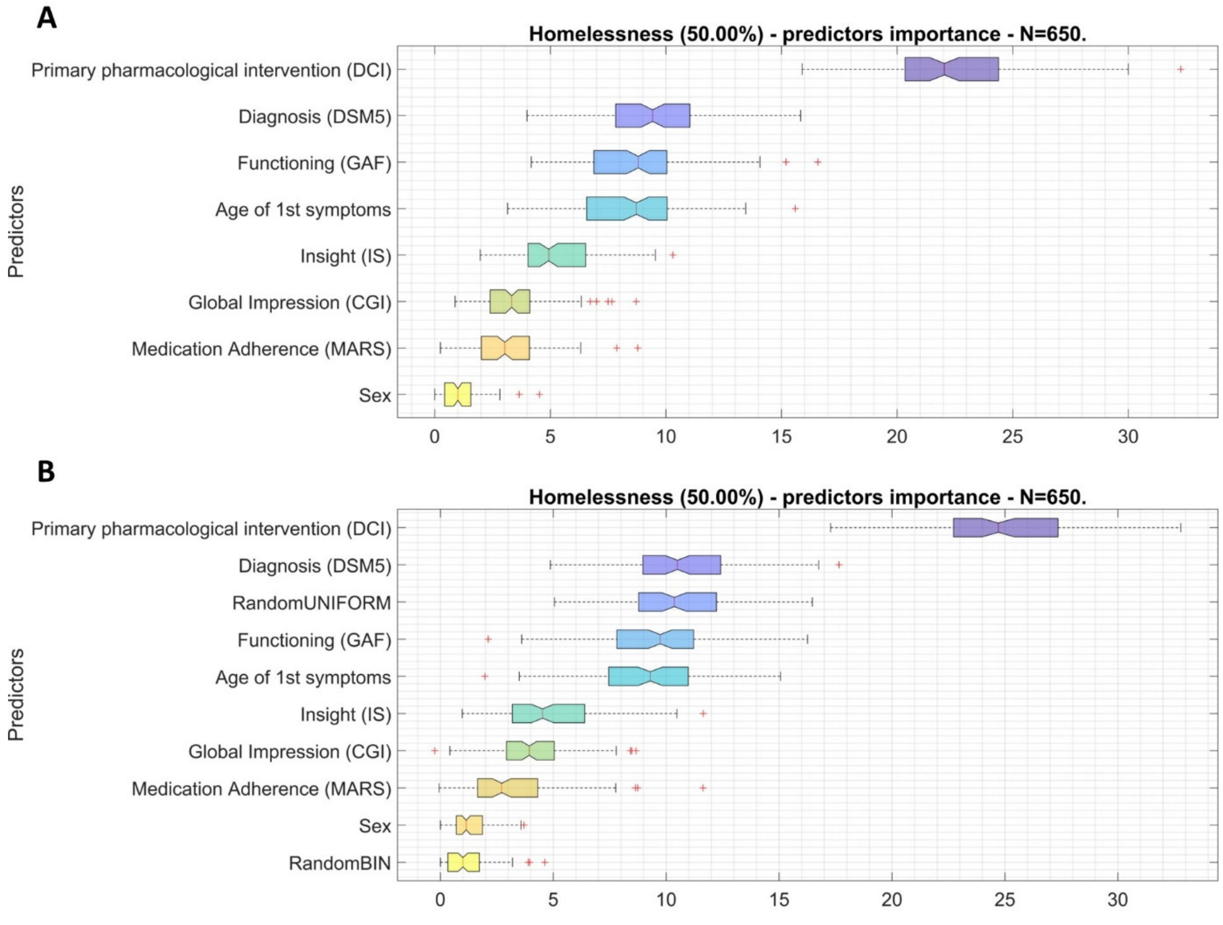

Figure S5

***Supplementary analysis 2** – Post-hoc analysis on the four clinical rating scales (GAF, CGI, MARS and BIS).*

A post-hoc analysis was performed to test whether there were significant differences between our two cohorts on the mean scores of the GAF, CGI, MARS and BIS clinical scales. Four Student's t tests, corrected for multiple comparisons by the Bonferroni correction, were performed (Fig. S6-A). Only the GAF and CGI scales showed significant effects, with lower GAF values and higher CGI values predicted in the population that had been confronted with homelessness (Fig. S6-B).

A

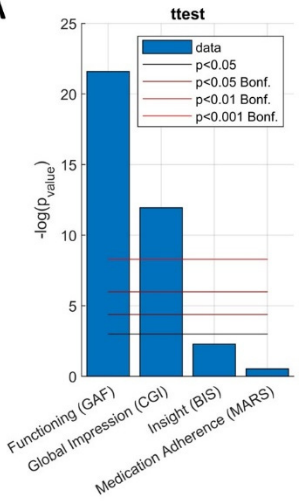

B

Homelessness	GAF [1-100] (higher is better)	CGI [1-7] (lower is better)
Yes	53,3 (σ=15,1)	4,28 (σ=1,12)
No	59,15 (σ=14,2)	3,95 (σ=1,12)
P-value (ttest)	<4,2x10^{-10}	<6,5x10^{-6}

Figure S6

Supplementary analysis 3 *– Post-hoc analyses on the variable "Age of the first symptoms".*

The variable "age of first symptoms" is suspected to have complex relationships with the prevalence of homelessness in a psychiatric population. Early onset of symptoms related to neurodevelopmental disorders and very late onset of symptoms are suspected to be 'protectives' with respect to syndromes with symptoms that appears during adolescence and adulthood, such as schizophrenia, bipolar disorders or depression.

For this analysis, the population was divided in 4 age groups of onset of the first symptoms: 0 to 15, 16 to 29, 30 to 39, and over 40 years old. Differences in prevalence of homelessness were analyzed using contingency tables and successive Pearson's chi-square tests. Population sizes of the four age groups are presented in figure S7-A (subjects who have been/being confronted with homelessness in red). The measured prevalences in S7-B and the corresponding Chi2 statistics and p-values in figure S7-C and S7-D, respectively. In our large multidiagnostic cohort, no significant relationship between age of the first symptoms and homelessness was found.

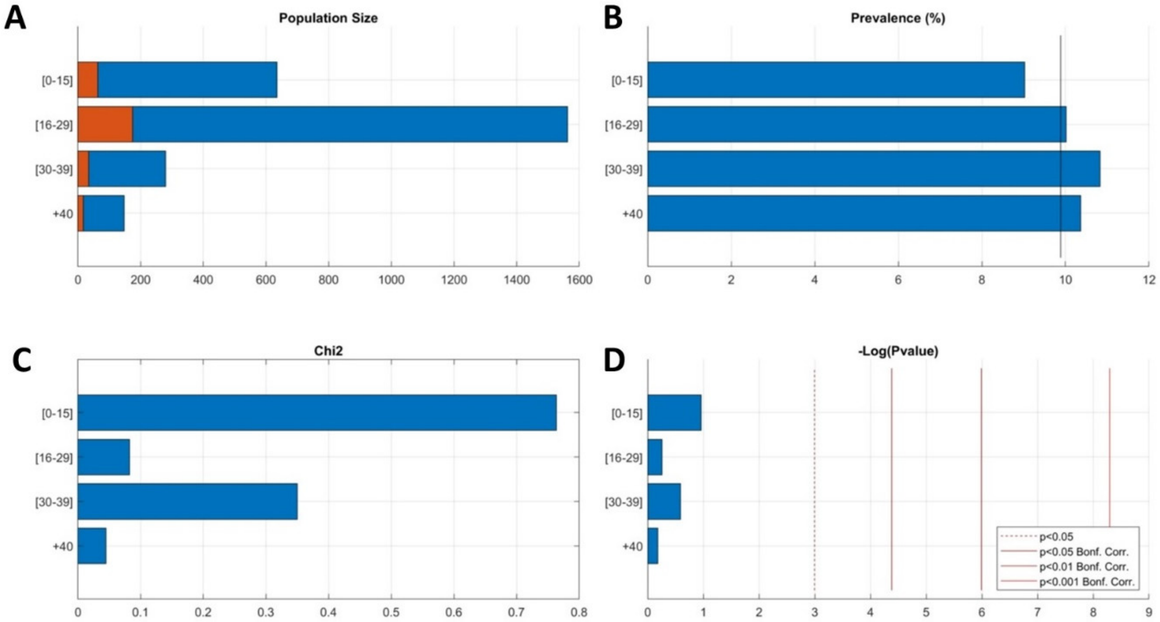

Figure S7
